# Regenerative fibroblasts derived from autologous skin tissue for the treatment of Sjögren’s syndrome: a case report

**DOI:** 10.3389/fimmu.2025.1529883

**Published:** 2025-01-27

**Authors:** Zhao-Xia Ma, Xing-Fei Wu, Li Cao, Cheng-Yan Jiao, Dai-Ping Ma, Yun-Hui Zhao, Zhi-Xing Yang, Min Hu

**Affiliations:** ^1^ Yunnan Key Laboratory for Basic Research on Bone and Joint Diseases, Kunming University, Kunming, Yunnan, China; ^2^ Production Department, Yunnan Jici Institute for Regenerative Medicine Co., Ltd, Kunming, Yunnan, China; ^3^ Research and Development Department, Shenzhen Zhendejici Pharmaceutical Research and Development Co., Ltd., Shenzhen, Guangdong, China

**Keywords:** case report, Sjögren’s syndrome, regenerative fibroblasts, purpura, cytokines

## Abstract

**Background:**

Sjögren’s syndrome (SS) is a systemic autoimmune disease, with major symptoms including dry mouth and dry eyes, for which there is no effective treatment. Recent studies have demonstrated that mesenchymal stem cells (MSCs) are effective in the treatment of SS, but the efficacy of allogeneic MSCs is affected by variability among different cell donors, and they are easily cleared by the immune system of the recipient. Autologous MSCs are one of the ideal options for the treatment of SS; however, their function decreases with age. Regenerative fibroblast (rFib) is a type of new MSC obtained through chemical reprogramming technology from skin fibroblasts. In this study, we report the safety and efficacy of intravenous infusion of autologous rFib in a volunteer with SS.

**Case report:**

In March 2021, the volunteer was diagnosed with SS due to positive anti-SSB antibodies, lymphocyte infiltration in the lip gland, dry eyes, and a large area of purpura in both lower limbs. From May 2021 to November 2022, she received allogeneic Umbilical cord mesenchymal stem cells (UCMSC) therapy (5.0 × 10^7^ UCMSCs per time, totaling 10 infusions), but her condition did not improve. In May 2023, the rFib for the volunteer was prepared, meeting the quality standard of T/CSCB0003-2021 Human Mesenchymal Stem Cells. Between October 2023 and June 2024, the volunteer received a total of 12 intravenous transfusions of autologous rFib. After the treatments, the volunteer experienced no recurrence of purpura in both lower limbs. Symptoms of dry mouth, dry eyes, and fatigue were relieved. ESR, B lymphocytes, rheumatoid factor IgM, and IgA declined, while the proportion of NK cells increased, and most of the cytokines returned to normal levels. *In vitro* experiments showed that rFib could significantly inhibit the proliferation of T lymphocytes after PHA stimulation. No adverse effects were associated with the use of rFib in the volunteer during the clinical trial.

**Summary:**

The results of this clinical trial indicate that intravenous injections of autologous rFib are both safe and effective for treating SS. Autologous rFib may be more suitable for treating autoimmune diseases than allogeneic MSCs.

## 1 Introduction

Sjögren’s syndrome (SS) is a systemic autoimmune disease that is typically characterized by symptoms such as dry mouth, dry eyes, fatigue, joint pain, muscle pain, and dental caries, among others (1, 2). In severe cases, SS can cause systemic complications, including rash, kidney damage, lung damage, and neuropathy (3–6). One of the most severe and life-threatening complications of SS is cryoglobulinemic vasculitis (7). Mesenchymal stem cells (MSCs), known for their immune regulatory and tissue repair functions, can directly influence T-cell differentiation and function by secreting immune regulatory factors. These cells particularly enhance the production of regulatory T cells (Tregs), thereby suppressing inflammation (8). Additionally, MSCs inhibit the activity of Th1 and Th17 cells, which are critical in the autoimmune response of SS (9). MSCs can also inhibit the proliferation of B cells and the production of antibodies, including autoantibodies, by directly interacting with B cells and modulating their signaling pathways, thereby reducing the autoimmune response (10). Additionally, MSCs are effective in repairing damaged tissues (11). However, allogeneic MSC treatment for SS presents several challenges. These include the risk of immune rejection following transplantation, which can lead to inflammation, as well as the variability in outcomes depending on different cell donors (12, 13). Therefore, autologous MSCs are an ideal choice for SS treatment. The patented technology developed by the laboratory of the first author (who is also a clinical trial volunteer in this paper) reprograms chemically induced skin fibroblasts into MSC-like cells, referred to as regenerative fibroblasts (rFib). This process preserves the cells’ genetic sequence and ensures their functional activity is unaffected by the health status of the volunteer (14). Safety evaluations have been completed for both single and multiple intravenous administrations of autologous rFib in monkeys. In this paper, despite treatment with UCMSC therapy, the volunteer’s condition continued to deteriorate, significantly impacting her work, life, and emotional well-being. Following a review by the medical ethics committee at the first author’s institution, the case was treated as that of a volunteer. Autologous rFib was used for SS treatment research in a compassionate use context. The results are reported as follows.

## 2 Case report

### 2.1 Volunteer information

The volunteer was pregnant in 2017 and frequently noticed small red spots on her feet. Since 2018, she has experienced dry mouth, dry eyes, and occasional red spots on her lower limbs. After speaking for 5 min without water, she struggles to pronounce words clearly. In March 2021, she sought medical attention due to widespread bruising in both lower limbs following prolonged standing. Tests revealed a positive anti-SSB antibody. A biopsy of the labial gland revealed focal infiltration of lymphocytes, with more than 50 cells/4 mm^2^. Dry eye indicators include a decreased lacrimal lake height, reduced tear film rupture time, and absence of active meibomian glands. These findings confirm a diagnosis of SS.

### 2.2 Therapeutic process

**Figure 1:**
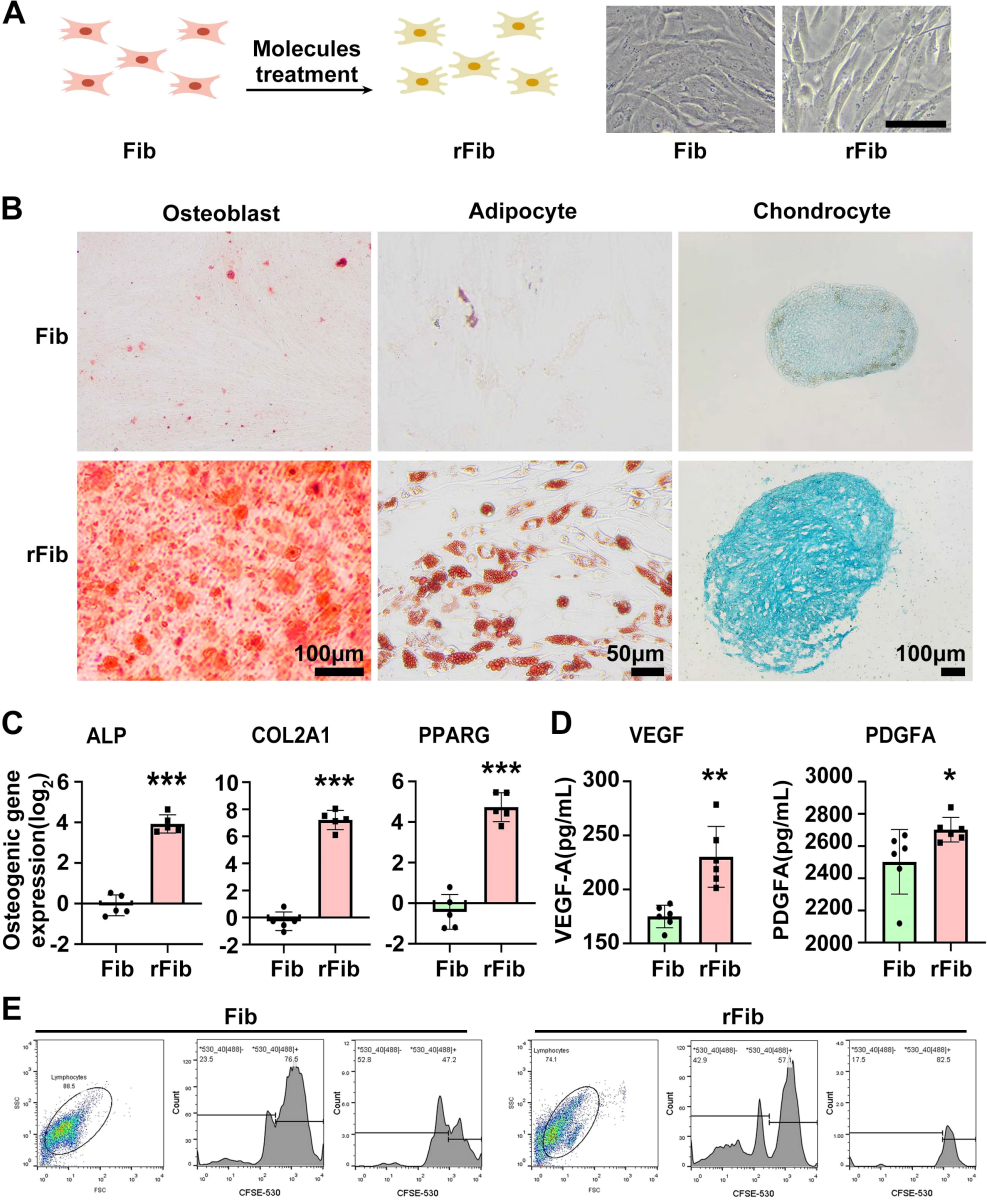
Preparation and functional identification of autologous rFib cells. **(A)** Preparation diagram of rFib. **(B, C)** rFib differentiation into osteoblasts, adipocytes, and chondrocytes. **(D)** Secretion of VEGF and PDGFA by rFib *in vitro*. **(E)** Immune regulatory functions of rFib. Data are presented as the mean ± SD (n = 5or6) with *P < 0.05, **P < 0.01, ***P < 0.001.

**Figure 2:**
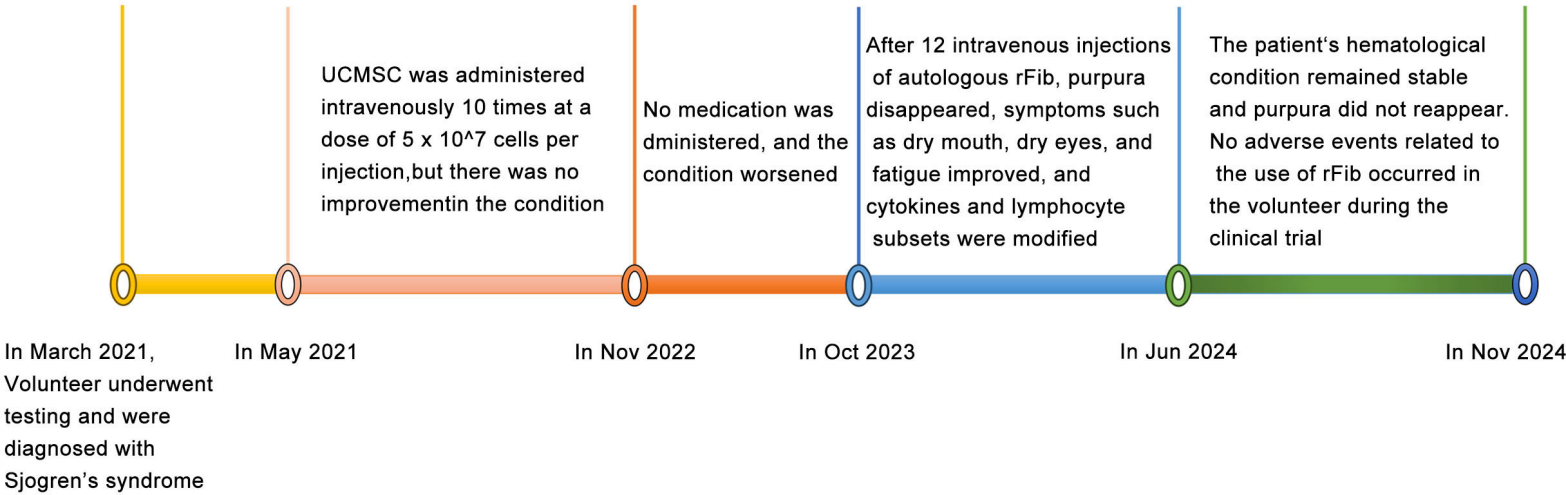
Volunteer’s diagnosed SS and its treatment process.

MSCs are known for their immunomodulatory functions and have been reported to alleviate SS to some extent (15, 16). With the volunteer’s consent, we followed the method by Liang et al. (17) to prepare and test the quality of UCMSCs. Between May 2021 and November 2022, UCMSCs (5 × 10^7^ cells per injection) were administered intravenously 10 times. Unfortunately, the volunteer’s symptoms, including purpura, dry mouth, and fatigue, did not improve, significantly affecting her work, daily life, and mental health. After a review by the Medical Ethics Committee at Kunming University and obtaining informed consent, skin fibroblasts were collected from the volunteer in May 2023 and reprogrammed into rFib using small molecule compounds, following the patented technology (US 11,674,122 B2) by Hu et al. (14) (**Figure 1A**). rFib can differentiate into osteoblasts, adipocytes, and chondrocytes (**Figures 1B, C**). It secretes vascular endothelial growth factor (VEGF) and platelet-derived growth factor A (PDGFA) *in vitro* (**Figure 1D**) and exhibits immune regulatory functions (**Figure 1E**). Following quality testing, the volunteer received 12 intravenous infusions of autologous rFib between October 2023 and June 2024. The first four times were administered weekly, while the last eight were scheduled every 4 weeks. For the initial seven infusions, the number of cells was 5 × 10^7^. Given the absence of adverse reactions during the clinical trial, the cell count was increased to 7.5 × 10^7^ for the final five infusions. **Figure 2** illustrates the treatment progression for this volunteer, including UCMSC and rFib.

### 2.3 Change of body condition

**Figure 3:**
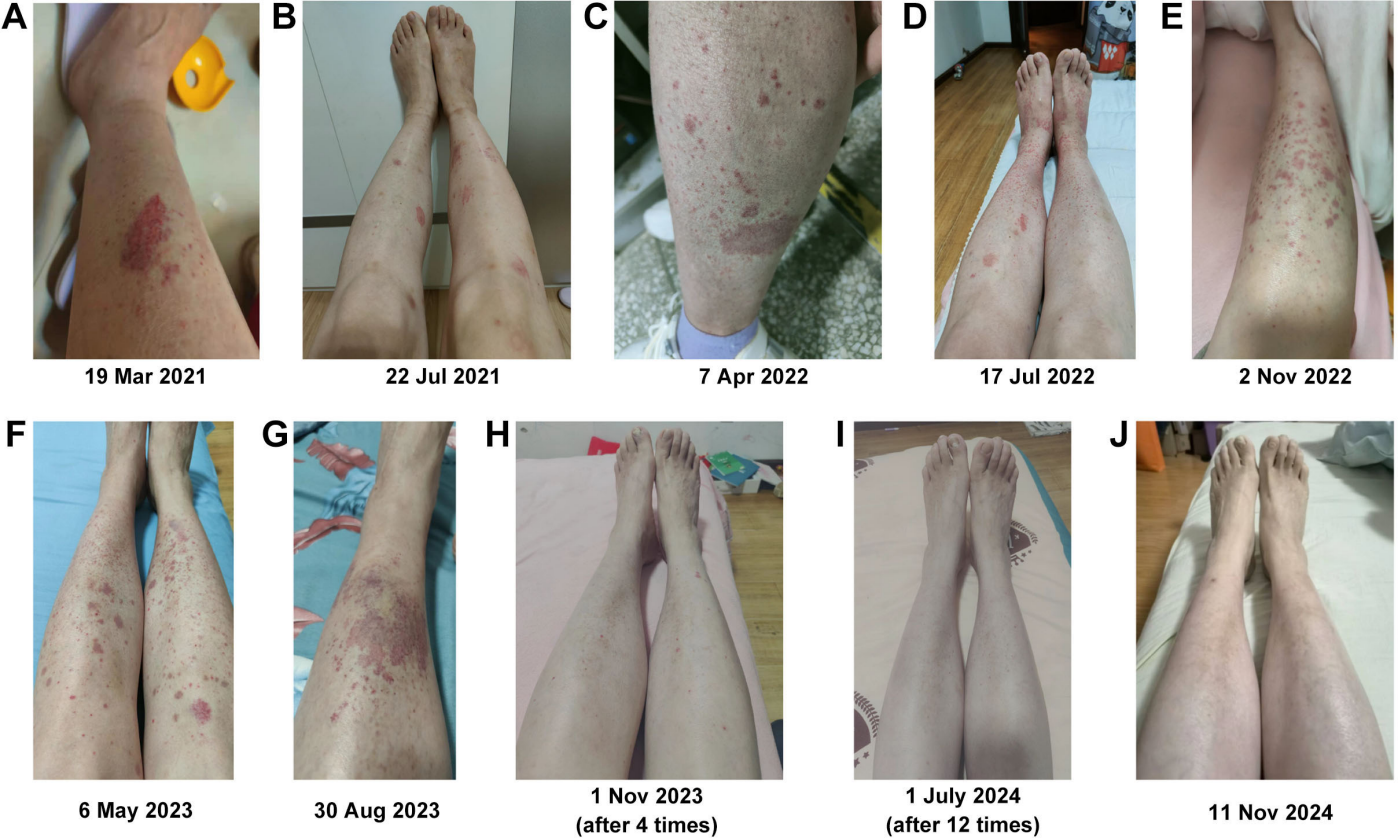
Changes in purpura in both lower limbs. **(A)** Before allogeneic UCMSC infusion. **(B)** After three intravenous allogeneic UCMSC infusions. **(C)** After six intravenous allogeneic UCMSC infusions. **(D)** After nine intravenous allogeneic UCMSC infusions. **(E)** Before 10 intravenous allogeneic UCMSC infusions. **(F)** After 10 intravenous allogeneic UCMSC infusions. **(G)** Before infusion of autologous rFib. **(H)** After four intravenous autologous rFib infusion. **(I)** After 12 intravenous autologous rFib infusion. **(J)** One year after the infusion of autologous rFib.

The volunteer was diagnosed with SS in March 2021, but her condition did not improve after UCMSC treatment. She experienced dry mouth and had to wake up in the middle of the night to drink water. She also had reduced tear production and general weakness, particularly in her lower limbs, which felt heavy. Climbing the stairs of the 10th floor was difficult, requiring two to three rest breaks during the journey, and she frequently felt tired. Purpura frequently appeared on both lower limbs following fatigue, prolonged standing, or extended sitting (**Figures 3A–G**). After four infusions of rFib, the volunteer experienced relief from dry mouth and dry eyes, and large areas of purpura rarely appeared on her lower limbs (**Figure 3H**). After 12 transfusions of rFib, the volunteer experienced an increase in saliva production, no longer needed to get up during the night to drink water, and had tears. She felt stronger overall, with relaxed legs, and was able to hike 5 km up a mountain without feeling tired afterward. Additionally, the purpura marks on her lower limbs had faded (**Figure 3I**). By November 2024, the purpura had completely disappeared from her lower limbs (**Figure 3J**).

### 2.4 Changes in serological indexes

Cytokines play an important role in the occurrence and development of SS (18, 19). Therefore, we measured 12 related cytokines in serum before rFib therapy, as well as again after four and 12 treatments. Before the use of autologous rFib, levels of all cytokines, except interferon-α (IFN-α) and tumor necrosis factor-α (TNF-α) were elevated, particularly the proinflammatory cytokines IL-1β, IL-6, and IL-17α, as well as the chemokine IL-8, which were more than 10 times higher than normal values. After four transfusions of rFib, the levels of IL-1β, IL-17α, and IL-8 decreased, while the levels of other cytokines increased compared to their levels before infusion. After 12 infusions of rFib, the values of seven cytokines (IFN-α, IFN-γ, IL-10, IL-1β, IL-5, IL-8, TNF-α) returned to normal levels, while the other five cytokines (IL-12p70, IL-17α, IL-2, IL-4, IL-6) remained elevated. However, the levels of the remaining cytokines were close to the normal range; notably, the level of IL-17α decreased from 199.63 to 9.76 pg/ml, which is only 0.45 pg/ml below the normal range (**Table 1**).

**Table 1:** The major clinical laboratory characteristics of the volunteer.

Items	Index (units)	Normal range	Before rFib cell infusion	After four cell infusions	After 12 cell infusions	Index changes
Cytokines	IL-10 (pg/ml)	< 8.051	**16.33**	34.86	**4.94**	**↓** (Return to normal)
	IL-12p70 (pg/ml)	< 6.024	15.23	29.89	12.19	**↓**
	IL-1β (pg/ml)	< 13.04	**233.62**	33.53	**6.74**	**↓** (Return to normal)
	IL-2 (pg/ml)	< 7.394	24.13	36.22	10.37	**↓**
	IL-4 (pg/ml)	< 7.541	40.97	27.79	10.06	**↓**
	IL-6 (pg/ml)	< 9.1	108.87	285.96	23.98	**↓**
	IL-8 (pg/ml)	< 20.98	**241.00**	196.61	**17.65**	**↓** (Return to normal)
	TNF(α) (pg/ml)	< 14.192	**11.55**	28.83	**9.68**	Normal
	IFN-α (pg/ml)	< 8.5	**2.18**	36.56	**4.9**	Normal
	IFN-γ (pg/ml)	< 11.25	**33.26**	89.17	**9.27**	**↓** (Return to normal)
	IL-5 (pg/ml)	< 6.398	**16.13**	67.51	**5.11**	**↓** (Return to normal)
	IL-17α (pg/ml)	< 9.31	199.63	139.06	9.76	**↓**
Lymphocyte subpopulation	CD3 (%)	56.5–85.5	62.90	64.40	61.54	Normal
	CD3+CD45+ (%)	56.0–85.0	62.80%	64.30	61.44	Normal
	CD3+CD4+CD45+ (%)	30.0–54.0	**28.60**	28.20	**30.45**	**↑** (Return to normal)
	CD3+CD8+CD45+ (%)	15.0–34.0	23.90	29.20	26.08	Normal
	CD4+CD8+ (%)	0–2	0.10	0.64	0.70	Normal
	CD4/CD8 (%)	1.0–2.0	1.20	0.97	1.17	Normal
	B lymphocyte (CD19+) (%)	7.3–18.2	**28.90**	24.30	**26.79**	**↓**
	NK cells (%)	8.1–25.6	**5.90**	9.60	**10.52**	**↑** (Return to normal)
ESR	ESR (mm/h)	0–20	36	31	25	**↓**
CRP	CRP (mg/L)	0–2.87	1.01	< 0.91	2.06	Normal
RF	rheumatoid factors IgA (U/ml)	0–15	**305.055**	375.829	**95.402**	**↓**
	rheumatoid factors IgG (U/ml)	0–25	13.03	12.848	37.665	**↑**
	rheumatoid factors IgM (U/ml)	0–25	**331.472**	191.032	**179.775**	**↓**
Immunoglobulin	Immunoglobulin G (g/L)	8.6–17.4	**25.3**	24.4	**25.2**	–
	Immunoglobulin A (g/L)	1.0–4.2	2.37	2.41	2.07	Normal
	Immunoglobulin M (g/L)	0.5–2.8	1.15	1.10	1.09	Normal
Complement	C3 (g/L)	0.9–1.8	1.13	1.11	–	Normal
	C4 (g/L)	0.1–0.4	0.29	0.26	–	Normal

The bold values in the table 1 highlight indicators that significantly changed before and after using 12 autologous rFib. Furthermore, immunoglobulin G indicates that the appearance and disappearance of purpura are not related to it. The symbol ↓ indicates a decrease, while the symbol ↑ signifies an increase.

SS is an autoimmune inflammatory disease characterized by the infiltration of lymphocytes into the salivary and lacrimal glands, which ultimately leads to gland destruction. The activation of T lymphocytes and B cells triggers the infiltration of inflammatory cells, resulting in glandular tissue damage and metabolic changes associated with SS (20). We analyzed peripheral blood lymphocyte subsets before the infusion of rFib and after four and 12 infusions. Before the infusion of rFib, the proportion of NK cells and CD3+CD4+CD45+ T lymphocytes was below the reference value, while the proportion of B lymphocytes was above the reference value. After four transfusions of rFib, the proportion of NK cells increased and returned to the normal reference range. The proportion of B lymphocytes decreased after 12 transfusions of rFib, while the proportion of NK cells increased further, and the proportion of CD3+CD4+CD45+ T lymphocytes increased, returning to the normal reference range (**Table 1**).

Patients with SS typically show elevated levels of erythrocyte sedimentation rate (ESR) and C-reactive protein (CRP) in their serum (21, 22). Consequently, we measured ESR and CRP levels before and after the injection of rFib. The results indicated that CRP remained within the normal range both before and after the infusion. In contrast, the initial ESR was 36 mm/h before the infusion of rFib, decreased to 31 mm/h after four infusions, and further decreased to 25 mm/h after 12 infusions of rFib (**Table 1**).

SS is an autoimmune disease characterized by the excessive production of autoantibodies, including rheumatoid factors (RF) (23). We similarly tested immunoglobulin and RF levels before and after the infusion of rFib. Before the infusion, RF A was 305.055 U/ml, and RF M was 331.472 U/ml. After four transfusions of rFib, RF A increased to 375.829 U/ml, while RF M decreased to 191.032 U/ml. After 12 transfusions of rFib, RF A decreased to 95.402 U/ml, while RF M decreased to 179.775 U/ml. These results are summarized in **Table 1**.

One of the most severe organ and life-threatening complications of SS is cryoglobulinemic vasculitis (7). To determine whether the purpura on the volunteer’s legs was related to the immunoglobulin and complement levels, we measured the serum levels of immunoglobulins A, G, and M, as well as complement levels 3 and 4, before and after rFib injection. The results indicated that, before the rFib injection, immunoglobulins A and M, as well as complement levels 3 and 4, were normal, while immunoglobulin G levels were elevated. However, after the rFib injection, immunoglobulin G levels remained unchanged, even as the purpura disappeared (**Table 1**).

### 2.5 Other indicators remain unchanged

We conducted several examinations, including antinuclear antibody detection, echocardiogram, and chest CT, both before and after the infusion of rFib. However, since these measurements did not show any changes, they are not included in the results.

## 3 Discussion

From 2018 to 2023, the volunteer experienced dry mouth, purpura in both lower limbs, and fatigue, which significantly affected her daily life, work, and mood. Since rFib is derived from the volunteer’s own body and has undergone GLP safety evaluation in preclinical studies, intravenous infusion of autologous rFib was conducted under compassionate use conditions.

Following autologous rFib treatment, a significant improvement was observed in the absence of widespread purpura. Symptoms such as dry mouth, fatigue, and difficulty walking briskly also improved greatly. Unfortunately, no ultrasound or lower limb skin biopsy was performed prior to the rFib cell infusion, nor was the saliva flow rate assessed. Consequently, the impact of these laboratory findings remains uncertain.

Laboratory serology results showed significant changes in cytokines, lymphocyte subsets, and erythrocyte sedimentation rate before and after rFib infusion. Abnormal cytokine secretion plays a significant role in the pathogenesis of SS, including the overexpression of proinflammatory factors such as TNF-α, IFN-γ, IL-1β, IL-6, and IL-17, alongside reduced expression of the anti-inflammatory factor TGF-β (24–26). Prior to the infusion of autologous rFib, the expression levels of proinflammatory cytokines IL-1β, IL-6, IL-17α, and the chemokine IL-8 were over 10 times higher than normal, consistent with previous reports (18, 27). After the infusion, the levels of highly expressed proinflammatory cytokines decreased to normal or near-normal levels. The histological features of SS include immune cell infiltration in the lacrimal and salivary glands, involving CD4+ Th cells, CD8+ cytotoxic T (Tc) cells, B cells, and plasma cells. CD4+ T cells predominate in the early stage of SS, whereas B cells or plasma cells become more dominant as the disease progresses (26, 28). Before the infusion of rFib, the volunteer’s serum exhibited a reduced proportion of NK cells and an increased proportion of B lymphocytes. Following the infusion, NK cell levels returned to normal, while B lymphocyte proportions decreased (**Table 1**). The ESR is a key inflammatory marker in SS volunteers (29). In this case, the ESR was 36 mm/h before the infusion of autogenic rFib, decreased to 31 mm/h after four transfusions, and further declined to 25 mm/h after 12 transfusions (**Table 1**), indicating that rFib cells can effectively reduce ESR.

The mechanism by which autologous rFib treats SS involves significant inhibition of PHA-stimulated lymphocyte proliferation, as demonstrated by our *in vitro* experimental results (**Figure 1E**). Following autologous rFib therapy, changes in cytokines and lymphoid subsets *in vivo* may be related to the immunomodulatory functions of rFib. The levels of PDGFA and VEGF increased in the rFib culture medium (**Figure 1D**). PDGF primarily facilitates angiogenesis and cell proliferation (30), while VEGF promotes angiogenesis and enhances tissue blood supply, potentially aiding in the functional recovery of glands. Moreover, VEGF plays a crucial role in regulating the inflammatory response and may influence the onset and progression of inflammation by modulating immune cell activity in the microenvironment (31). In a clinical study, Pan et al. used an anti-VEGF antibody to treat a volunteer with bilateral cystoid macular edema complicated by anaphylactoid purpura (32). We speculate that the disappearance of purpura in both lower limbs of the volunteer may be related to these two cytokines rather than serum immunoglobulin and complement levels (**Table 1**).

Previous studies indicate that allogeneic UCMSCs can alleviate dryness symptoms in both mouse models and SS patients (8, 9). However, the volunteer with Sjögren’s syndrome in this study did not respond to allogeneic UCMSCs. We speculate that several factors may contribute to this outcome. First, UCMSCs are derived from various umbilical cord donors, each with a unique biological backgrounds. As a result, UCMSCs from different donors may produce varying therapeutic effects on SS. Secondly, while UCMSCs have low immunogenicity, they are eventually cleared after allotransplantation due to immune rejection by the host (SS patients), leading to short-lived and limited efficacy. The survival duration of UCMSCs in the host (SS patient) depends on both the immunogenicity of the UCMSCs and the immune status of the host.

Our findings suggest that autologous rFib may help treat SS by modulating the immune system, reducing inflammatory cytokine production, and lowering the erythrocyte sedimentation rate. Although the volunteer’s physical condition has significantly improved, the limited treatment duration has resulted in few studies on the mechanism of autologous rFib in treating SS. In the future, we will continue to monitor the volunteer’s health changes and use mouse models to investigate the mechanism of autologous rFib in treating SS.

## 4 Conclusion

This clinical trial demonstrates that intravenous injection of autologous rFib is safe and effective for treating SS, potentially due to its immunomodulatory function. This is the first case in which autologous rFib cells have been used to treat SS. Our data are limited to purpura and serological indicators from the lower limbs, with no involvement of lung tissue, joints, or other lesions in this case. If future volunteers present with more severe symptoms, we can consider treating them with autologous rFib as well.

## Data Availability

The original contributions presented in the study are included in the article/supplementary material. Further inquiries can be directed to the corresponding author.
